# Viral Phrenology

**DOI:** 10.3390/v13112191

**Published:** 2021-10-30

**Authors:** David P. Wilson, Danielle A. Roof

**Affiliations:** Department of Physics, Kalamazoo College, Kalamazoo, MI 49006, USA; droof1@tulane.edu

**Keywords:** Baltimore Classification, virus genome, Triangulation number, protruding features, spherical virus, point arrays, surface modifications, virus-like particle, VLP, nanomedicine

## Abstract

We introduce Viral Phrenology, a new scheme for understanding the genomic composition of spherical viruses based on the locations of their structural protrusions. We used icosahedral point arrays to classify 135 distinct viral capsids collected from over 600 capsids available in the VIPERdb. Using gauge points of point arrays, we found 149 unique structural protrusions. We then show how to use the locations of these protrusions to determine the genetic composition of the virus. We then show that ssDNA, dsDNA, dsRNA and ssRNA viruses use different arrangements for distributing their protrusions. We also found that Triangulation number is also partially dependent on the structural protrusions. This analysis begins to tie together Baltimore Classification and Triangulation number using point arrays.

## 1. Introduction

We studied 135 distinct spherical viruses taken from VIPERdb [1] and found that the locations of their structural protrusions often indicated their genetic composition. We refer to this method of determining a virus’s genetic composition by examining the placement of its protrusions as Viral Phrenology. The structural protrusions were found by classifying the viruses using our modified fitting methods [2,3] for icosahedral point arrays [4,5]. Point arrays provide highly specific geometric constraints on the arrangement of viral proteins, and all spherical viruses studied so far conform to one or more of these arrays [3,4,5,6,7,8,9,10,11,12,13,14]. We have previously shown that all protruding features of spherical viruses are located on the gauge points of these arrays, though it was not yet known that these locations also indicated the genomic composition. The gauge points determine the overall radial scaling of a point array and are all located on the 15 icosahedral great circles which subtend neighboring symmetry axes in the asymmetric unit [2,3]. We also show that not all gauge points are used for both DNA and RNA viruses. Additionally, we see that the Triangulation number [15] is also semi-indicated by the location of protrusions.

Spherical virus capsids have icosahedral symmetry, and are classified by the Triangulation (T)-number, which posits that viral capsid proteins are arranged in nearly identical chemical environments, known as quasi-equivalence. In general, T-number specifies the number of identical capsid proteins (60T), that there are 12 pentameric units and 10(T−1) hexameric units. There are a number of viruses that make exceptions to these rules, though remarkable they still ascribe to the architectures prescribed by the T-number. For example, SV40 [16] has the architecture of T7d, though it is only composed of 360 proteins, rather than 420 and it is made entirely from pentamers. Remarkably, the virus maintains icosahedral symmetry with pentamers located at each hexamer location. In addition, there are pseudo-T (pT) number viruses, which mimic larger T-number architectures using either different proteins or fewer multi-domain proteins [17]. For example, Human Rhinovirus 16 is a pT3 virus that is composed of only 60 proteins, yet it has a T3 architecture.

While spherical viruses have been well described by T-number, this classification does not provide any connection to the Baltimore Classification (BC) system [18], which organizes viruses into seven groups based on how they use mRNA in their replication cycle [19]. While cellular life is restricted to storing its genetic information within dsDNA, viruses are much more diverse. Viruses utilize dsDNA (BC I and BC VII), ssDNA (BC II), dsRNA (BC III) and ssRNA (BC IV, V and VI) genomes. To our knowledge, this study is the first to reveal the connection between the geometric arrangement of protruding features and the genetic composition of the virus. Given that the restrictions of point arrays are so specific, this understanding could inform evolution and mutation studies, development of virus-like particles (VLPs), and other potential therapeutic virus treatments.

In this paper, we present our new finding that the location of protruding features strongly indicates the genetic composition of many viruses. In 2016, we showed that all spherical viruses use a common set of 21 gauge points in the asymmetric unit for their protruding features [2]. This work further refines this result to show that entire sections of gauge points are only used by RNA or DNA viruses and some are not used at all. These gauge points come from the set of point arrays constructed through affine extensions of icosahedral polyhedra [2,3,4,5]. We previously demonstrated that point arrays indicate important geometric locations which should only be modified with care [3]; including the surprising immutability of the non-structural surface loops of the bacteriophage MS2 [20,21], the unexpected limited reactivity of solvent-exposed surface lysines in CPMV [22,23] and the non-quasiequivalent flexibility of the HBV dimers [24]. We present our new findings that the locations of protrusions indicate genetic composition, referred to mirthfully as Viral Phrenology. These results are displayed through 21 unique capsid images and reveal an entirely new way of considering virus evolution and modifications.

## 2. Materials and Methods

We used icosahedral point arrays to classify a library of 135 distinct spherical capsids [3]. We then studied the connections between key features of point arrays, including their gauge point locations and the standard characterizations of spherical viruses, including Triangulation number (T-number) and Baltimore Classification (BC). We identified key structural protrusions as those which were used to determine the point array classifications.

### 2.1. Virus Library

We gathered over 600 spherical virus structures from those deposited in VIPERdb [1]. These structures are determined in a variety of manners, including X-ray crystallography, cryoEM and model fits to cryoEM. We then narrowed this list down, by keeping each unique family, genus, Triangulation number (T-number) and Baltimore Classification (BC) combination. We then chose from the remaining structures the ones with the best resolution; when ties existed, we kept both structures; see Appendix A for the complete library. The result of this filtering lead to 135 spherical virus capsids, ranging from T1 to T169 and also included pseudo T-numbers. The genomic composition and T-number for this library is shown in Table 1. This criteria also included some viruses structures which were bound small molecules or complexed with antibodies. The goal of this methodology was to create a diverse data set, and in total there are 50 distinct Virus families and 105 distinct genera. We decided to focus only on the genomic composition, rather than the mRNA pathways specified for simplicity. To our knowledge, there are no spherical viruses with BC VI in the VIPERdb [1]; so, in general, when we say dsDNA, we mean BC I, ssDNA is BC II, etc. The only ambiguity exists for circular-dsDNA genome with an RNA-phase, as in HBV which is BC VII. Lastly, not all capsids found in VIPERdb [1] had an identified genome.

### 2.2. Point Arrays

**Figure 1:**
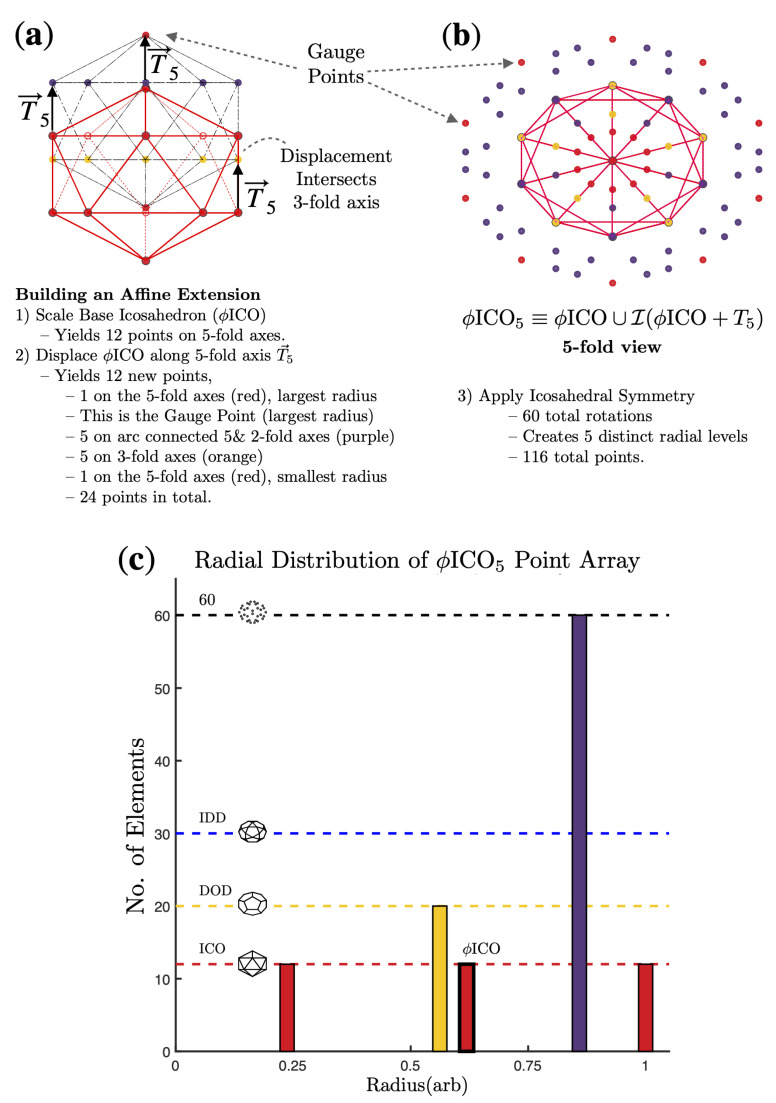
Point arrays are formed by performing an affine extension on the vertices of icosahedral polyhedra and then reapplying the rotation symmetry. The goal of this construction is to create representations of icosahedral symmetry at multiple radial levels in a mathematically consistent way, avoiding the formation of free groups [3,4,5]. We present all of our point array elements with the same color-mixing scheme. Red is 5-fold, blue is 2-fold, purple is directly between them. The 3-folds are yellow and between 3–5 is orange. Between 2–3 is green. Finally, any points not on the symmetry axes or the arcs connecting them are black. (**a**) The base icosahedron ICO is scaled by ϕ and then displaced along the 5-fold axis $\left({\vec{T}}_{5}\right)$. This creates 12 new points, at 4 different radii. (**b**) We now apply icosahedral symmetry to the translated vertices, creating a new cloud of points which surround the base icosahedron. While the icosahedral group has 60 rotations, each translated point does not generate 60 new points. For instance, the gauge point, which is located on the 5-fold axis only yields 12 unique points upon application of the rotations. (**c**) The point array $\phi {\mathrm{ICO}}_{5}$ consists of 5 different radial levels with a gauge point on the 5-fold axes. The entire point array will be scaled so that the gauge points match the radius of the center of mass of the capsids protrusions [2]. The point cloud can be visualized as an outer icosahedron with a layer of 60 points between the 5 and 2 symmetry axes, along the face of the icosahedron, though at lower radius. Then inside of this surface is another icosahedron (base, r∼0.64) that encloses a dodecahedron (DOD), which finally encloses a smaller icosahedron, at 24% of the radius of the gauge points.

**Figure 2:**
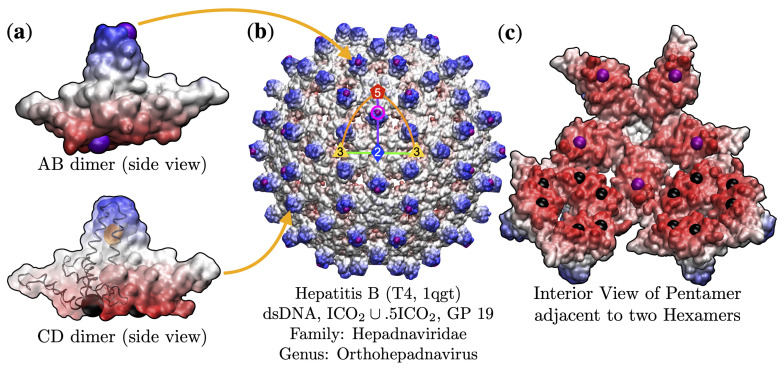
(**a**) The Hepatitis B capsid (HBV, T4, 1qgt [26]) is composed of 240 chemically-identical proteins, denoted chains A, B, C and D. These protein chains are arranged in two distinct dimers (AB and CD). According to point arrays, the AB dimer is constrained at the top (chain A and B, purple) and at the bottom (chain B only, purple). The CD dimer is constrained midway up the alpha helices (Both C D, orange) and then individually at the bottom of the C and D proteins (black). (**b**) These 120 dimers are arranged with icosahedral symmetry, leading to 12 pentamer and 30 hexamer subunits. In general, there are no covalent bonds between viral capsid proteins, including these dimers. Therefore other effects, such as hydrophobic and electrostatic interactions are required for stabilization [27,28,29]. Quasi-equivalence through icosahedral symmetry is the mechanism that ensures these interactions are plentiful and dispersed enough for capsid stability [15]. In the case HBV, extensive molecular dynamics simulations have shown these dimers to have different vibrational frequencies [24]. This difference is consistent with point arrays, as the AB dimer is constrained at the top and bottom, and the CD dimer is constrained at the middle and bottom. We have highlighted the C-chain protein backbone, which is constrained at two points to each side of the point array element (orange). The full HBV capsid, with the asymmetric unit (AU) highlighted between the 5-,2- and 3-fold axes. The AB dimers protrude between the 5- and 2-fold axes at gauge point 19 (pink circle). This gauge point determines nearly all of the other interior points, indicating that having protruding features at these locations puts constraints on the entire interior structure. None of these constraints are required by icosahedral symmetry. Gauge point 19 imposes inflexible restrictions on the choice of point arrays, either ${\mathrm{ICO}}_{2}$ or ${\mathrm{IDD}}_{5}$, are the only two point arrays available to this gauge point [2,3]. These are known as sister arrays, and are identical except for a single radial level and without more coordinate information on the genome, are indistinguishable at this level [3]. Each of these point arrays specify restrictions on three of the four protein chains A, C and D. (**c**) The interior view of a pentamer and two hexamer subunits. We see that the point arrays put constraints on the interior of the hexamer (black) and pentamer (purple). The two point arrays available to GP 19, ${\mathrm{ICO}}_{2}$ and ${\mathrm{IDD}}_{5}$ specify all the points shown in (**a**–**c**), except the purple points seen the interior pentamer in (**c**). These purple points comes from the union of ${\mathrm{ICO}}_{2}\cup 5{\mathrm{ICO}}_{2}$. This array imposes a restriction on chain B and is therefore the best-fitting point array. There is not a similar union available for ${\mathrm{IDD}}_{5}$. It is important to note that none of the points of point array ${\mathrm{ICO}}_{2}\cup 5{\mathrm{ICO}}_{2}$ can be removed or moved, there is only 1 degree of freedom, the overall radial scaling [3].

We classify viruses according to their best-fitting icosahedral point array(s) [4,5] using our fitness criteria introduced in [2,3]. These point arrays prescribe specific geometric constraints on viral capsids and their genetic material. These constraints are radially-distributed representations of icosahedral rotation symmetry which set limits on the angular orientation and distribution of virus capsid proteins; see Figure 1. While in principle, viruses could conform to multiple point arrays, in practice we find most viruses conform to only one or two arrays [3]. This classification is powerful, as it indicates geometric locations where viruses need to be modified with care, as well as other potential geometric locations where modifications should be relatively easy to accomplish. This classification is purely geometric in nature and does not take into account local surface chemistry, steric hindrance, or solvent accessibility. The classification can also help explain structural differences in the capsids, such as differences in vibrational modes of the dimers of HBV; see Figure 2.

Icosahedral point arrays offer a novel tool for analyzing viruses [2,4,5]. We have shown previously that amino acids near these geometric locations are likely critical to capsid stability and that many modifications, from amino acid substitution to ligand attachment are either prohibited or requires more care than expected by standard analysis such as steric hindrance and solvent accessibility [3]. Point arrays are constructed by applying an affine extension to a base icosahedron (ICO), dodecahedron (DOD) or icosadodecahedron (IDD) along the 2-, 3- or 5-fold symmetry axes direction then re-applying icosahedral symmetry. Each affine extension is characterized by the scale length of the base, in terms of the golden ratio ϕ and there are 55 different single base arrays [3,4,5]. For example, $\phi {\mathrm{ICO}}_{2}\equiv \phi \mathrm{ICO}\cup I\left(\phi \mathrm{ICO}+{\vec{T}}_{2}\right)$, where I represents applying the 60 rotations of the icosahedral group. This point array has a single gauge point, GP 21. Each array has a single free-parameter, the overall radial scaling. Point arrays with the same affine extension can be combined to form larger point arrays [3,4,5]. Many of our virus fits combine multiple point arrays.

**Figure 3:**
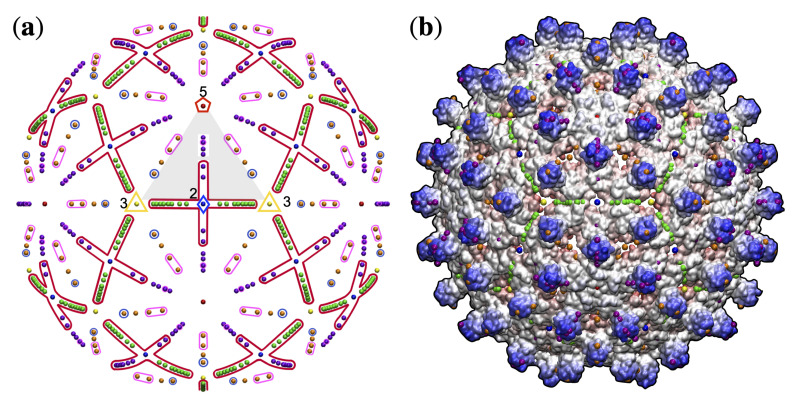
(**a**) The gauge points of the point arrays [2,3,4,5] are found on the icosahedral great circles which subtend neighboring symmetry axes, and define the Asymmetric Unit (AU), shown in grey. The AU is a 1/60th representative section of the full icosahedral capsid and there are 21 unique gauge points within it. These points are all colored based on where they are relative to the symmetry axes and the great circles they connect; 5-folds are red, 3-folds are yellow, 2-folds are blue, then purple is between 5 (red) and 2 (blue), green is between 2 (blue) and 3 (yellow), orange is between 5 (red) and 3 (yellow), finally black points are not on great circles, instead they are found anywhere else and are referred to as bulk points. The angular locations of these points are shown to scale. By analyzing the structural protrusions and the genomic composition of viruses, we found that only RNA viruses are found to use the encircled red region (⊥), and also dominate areas encircled with pink. Finally, only ssDNA viruses use the encircled blue region (near 3-fold axes). (**b**) The gauge points arranged atop HBV (T4, 1qgt) virus. There are two potential structural protrusions within the AU, located along the 5–2 arc (purple) and the 3–5 arc (orange). We find that only the point arrays corresponding to purple protrusion GP 19 have corresponding arrays which meet the criteria for a proper fit [3], so there is only one structural protrusion in HBV.

We have shown previously that all spherical protrusions are found on, or very close to the gauge points [2]. Our point array classification scheme begins by determining the center of mass for each viral protrusion, and then determining the gauge points nearest to them, based on an angular cutoff [2]; see Figure 3. We then scale all point arrays associated with these gauge points and optimize their RMSD fit [3]. In principle, a virus may have multiple protruding features on different gauge points. We refer to protruding features with gauge points that correspond to best-fitting point arrays as structural protrusions; that is their specific locations determine almost all of the internal structural constraints. The location of the gauge points determine the radial size of the point array and limit the possible interior arrays, imposing strong restrictions on the relative placement of capsid proteins at each radial level [3]; see Figure 3. It is also important to note, each gauge point not located on an icosahedral symmetry axes (2,3,5) has only 2 point arrays associated with it, so the locations of the gauge points place strong restrictions on the arrangement of capsid proteins. Our RMSD measure is found by

(1) $$\mathrm{RMSD}={\left(\frac{\sum_{i=1}^{N}m_{i}p_{i}d_{i}^{2}}{\sum_{i=1}^{N}m_{i}p_{i}}\right)}^{1/2}$$

where $d_{i}$ is the minimum distance from the $i^{\mathrm{th}}$ point array element to the nearest protein surface(s) or genome (if available) and $p_{i}$ is the number of different proteins near the point *i* (e.g., 5 for a point on a 5-fold axes or 2 if two proteins are equidistant from the same point). Finally, $m_{i}$ is the number of times the point appears in the full point array (i.e., 12, 20, 30 or 60) and *N* is the total number of elements in the point array.

After we have computed all the RMSD fits of point arrays which match the gauge points, we then decide which array(s) are the best fit [3]. In general, a better-fitting point array has at least 0.5 Å lower RMSD, has at least one point per protein and more overall points of contact with the capsid proteins. When multiple arrays meet this criteria, they are listed as well.

**Figure 4:**
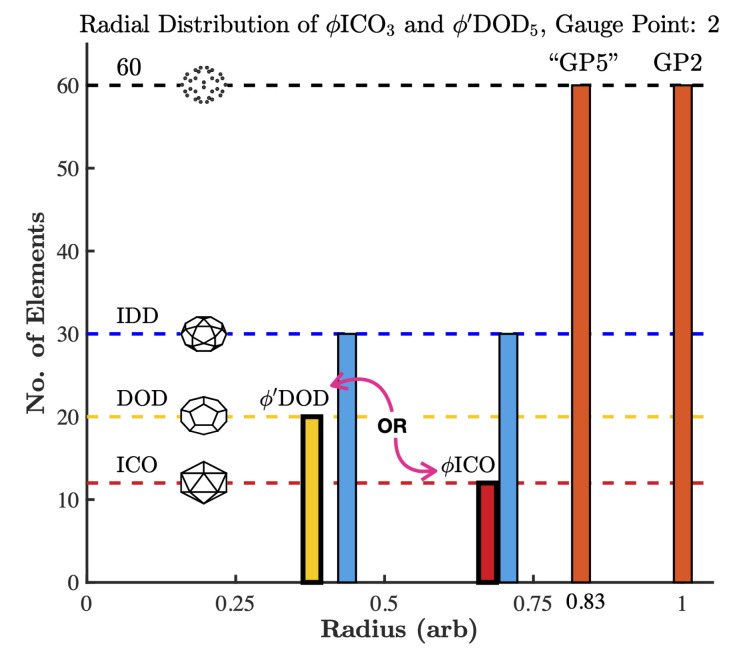

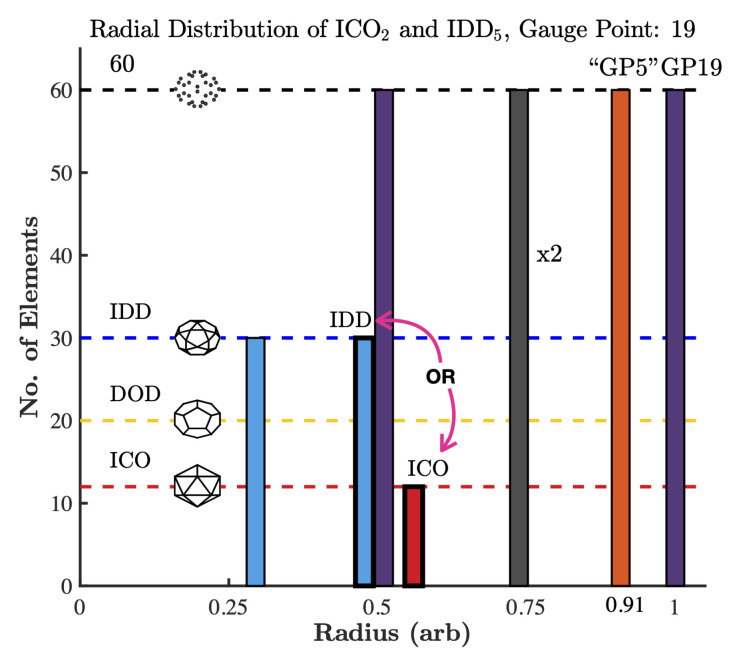
Gauge points impose a set of geometric constraints at different radii and angular locations throughout the capsid and genome. Here, we present 4 of the 55 base point arrays [3,4,5]. (**top**) Gauge point 2, located along the 5–3 great circle (orange), is created by $\phi^{\prime }{\mathrm{DOD}}_{5}$ (200 points) and $\phi {\mathrm{ICO}}_{3}$ (192 points). There are two sister arrays for every gauge point and these produce nearly identical point arrays, with only their base being different (outlined in bold), either DOD or ICO. Here, we see that if a protruding features is located at gauge point 2, then there must be another material boundary at 83% of that radius located at the same angular location as gauge point 5, though this point is not considered a gauge point because it is not the most radially distal. Then another boundary at the 2-fold axes (IDD) at 71% of the gauge point radius and so on. Most of the structures we have studied only consider the protein capsid; however, the genome would also be subject to these constraints. (**bottom**) Gauge point 19, located along the 5–2 great circle (purple), is created by ${\mathrm{ICO}}_{2}$ (342 points) and ${\mathrm{IDD}}_{5}$ (360 points). Here, we see that if a protruding feature is found at GP 19, then the next material boundary must be located at 91% of this radius, at the same angular location of GP 5. Then at 74% of this radius, there is a bulk constraint, 120 points in total; see Figure 3.

While some protrusions are known to play several important biological functions in gaining entry to new cells, as with herpes [30] and myonecrosis [31], the fact that they also constrain the structure of the interior of the capsid was a surprise [2]. In 2016, we discovered that all known protruding features of spherical viruses were located on 21 gauge points in the Asymmetric Unit (AU) [2]; see Figure 3. These gauge points were found by analyzing the distinct set of geometric locations determined specified by icosahedral point arrays. Two examples of the minimum level of constraints placed on a virus due to the location of its protruding features and thus gauge points can be seen in Figure 4. While point arrays dictate critical locations for viruses, they do not fully specify their structures. A summary of the number of angular and radial constraints based upon gauge point can be found in Table 2. The strict adherence of all known spherical virus protrusions to these locations suggest that they likely play a critical structural role in the stability of viruses. Some viruses, such as the bacteriophage MS2, adhere so strongly to these constraints that seemingly innocuous changes to their protruding features cause their virus structure to destabilize [20,21]. Nearly every virus we have characterized has material boundaries within a few angstroms of each of these points in the arrays corresponding to their gauge points.

## 3. Results

We determined the best-fitting point arrays [3] for 135 distinct viruses. We found 149 unique protrusions which determined the gauge points for these point array fits; which we classify as key structural protrusions. Furthermore, we want to understand why protruding features of viruses play such a critical role in the possible structural determination of the arrangements of proteins and even genetic material contained within the virus. There were 121 capsids which had a single best-fitting point array, and 14 which had two best-fitting point arrays, none of the capsids in our study had 3 or more best-fitting point arrays. Those capsids with two arrays, also had two different gauge points, leading to 14 more total protrusions than capsids studied. There are 38 dsDNA capsids, of which 34 have a single structural protrusion, 15 ssDNA capsids, of which 14 have a single structural protrusion, 17 dsRNA capsids, of which 14 have a single structural protrusion and 61 ssRNA capsids, with 55 having a single structural protrusion; see Table 1.

We found that, in general, there is no straightforward connection between T-number and Baltimore Classification of spherical viruses, in agreement with Louten [18]. There are, however, some interesting results to note as we found that nearly all ssDNA capsids (BC II) are T1 (87%), and most of the ssRNA capsids (70%) are T3 or pT3. In addition, most of the T3 capsids are only composed of ssRNA (87%), whereas T7, T16, and larger T-number capsids were found to only contain dsDNA. In summary, knowing the T-number provides limited information on the genome, as does only knowing the genome provide little information on the T-number. We only found two T9 structures, and three (pT169, T169), which may indicate a slight disagreement with the evolutionary representations suggested in [25], which stated that T9 should be well represented in viruses. We did not find any T12 or T19 capsids, though T27 and T169 were present.

### 3.1. Gauge Point vs. Triangulation Number

**Figure 5:**
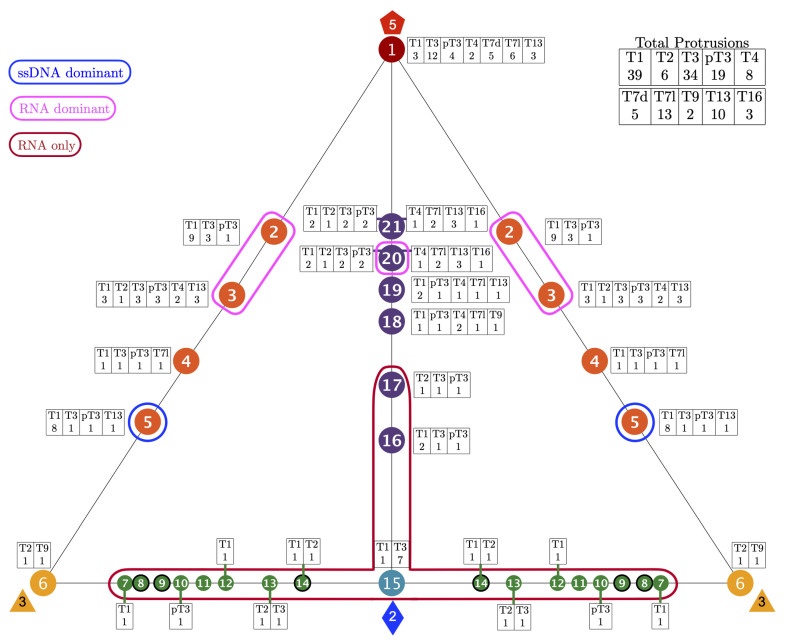
The 149 distinct structural protrusions and their corresponding T-numbers mapped onto the 21 unique gauge points of the Asymmetric unit (AU) [2]. The total number of protrusions for each T-number is shown in the upper right. The region encircled in red, GP 7 to GP 17 was found to only have RNA viruses with a T-number from 1 to 3. The regions encircles in pink (GP 2, 3 and 20) are dominated by RNA viruses (71% or more). Gauge point 5 is encircled in blue and primarily used by T1 capsid with ssDNA. The region between the 3-fold and 2-fold axes, GP 7 to 14 is not heavily used by any viruses. Overall, T-number is not strongly predicted by gauge point location. There are 10 viral protrusions belonging to T21 and larger capsids not shown here. All of these capsids used GP 1 or GP 21. The angular locations of the gauge points are shown to scale on a flat AU face. There is a sizeable gap between GP 1 and GP 2 and GP 21, as there is between GP 5 and GP 6.

We present the relationship between Triangulation number and gauge points in Table 3 and Figure 5. Overall, T-number and gauge points are independent though a few points stick out. GP 2 and 5 are primarily used by T1 capsids, 9 of 13 protrusions and 8 of 11 protrusions, respectively. GP 15 (2-fold axes) is primarily used by T3 capsids with 7 of 8 protrusions. We also found that few viral protrusions are found between the 3- and 2-fold axes, GP 6 to 15, though GP 15 has 8 capsids. In general, knowing the location of a structural protrusion does not strongly indicate the T-number. Capsids with a T-number greater than 16 all have a single structural protrusion located on GP 1 (5-fold) or GP 21.

### 3.2. Gauge Point vs. Genome

**Figure 6:**
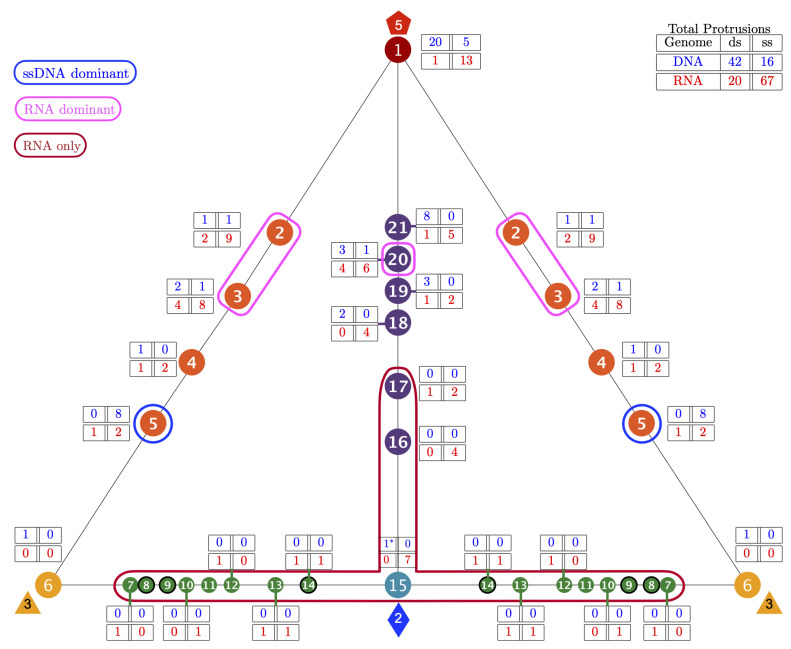
The 149 distinct structural protrusions and their corresponding genomes mapped onto the 21 unique gauge points of the Asymmetric unit (AU) [2]. The total number of protrusions for each genome is shown in the upper right. The region encircled in red, GP 7 to GP 17 was found to only have RNA viruses (Baltimore Classifications III, IV, and V). There is one technical exception to this observation, HBV forms a mixture of the T3 (5%) and a T4 (95%) capsids. The T3 capsid (6bvn) has two structural protrusions at GP 3 and GP 15; however, the T3 capsid is too small to package the DNA genome inside [32,33]. The regions encircles in pink (GP 2,3 and 20) are dominated by RNA viruses (71% or more). Gauge point 5 is encircled in blue and primarily used by T1 capsid with ssDNA (73%). Gauge points 1, 4, 18, 19 and 21 are equally utilized by RNA and DNA viruses. This visualization elucidates the new utility of Viral Phrenology, as a predictor of genome composition based on location of protrusions.

We present the relationship between genomic composition and gauge points in Table 4 and Figure 6. Overall, we find a strong relationship between the location of protrusions and genome composition. The most dramatic result was that only RNA viruses use gauge points 7 to 17, which we refer to as the upside down T/Up Tack (⊥) region; see Figure 5 and Figure 6. We also see that RNA capsids protrusions dominate GP 2 (85%), GP 3 (80.0%) and GP 20 (71%). Capsids with ssDNA are also clearly dominant at GP 5 (73%). There is also a clear relationship between dsDNA viruses between GP 1 and GP 21, representing 28 of 42 protrusions from 31 of the 38 dsDNA capsids. We also find that 13 of the 16 ssDNA protrusions are found between GP 1 (5-fold) and GP 5, accounting for 13 of the 15 ssDNA capsids; see Table 4 and Figure 6. Viruses with ssRNA compositions were spread all around the gauge points, with the largest number, 13 of 67 protrusions being found at GP 1. Similarly dsRNA capsids use a range of protruding feature locations. There were also some gauge points used by all genomes, GP 1, 2, 3 and 20; see Figure 6.

### 3.3. Gauge Points 1 to 6

**Figure 7:**
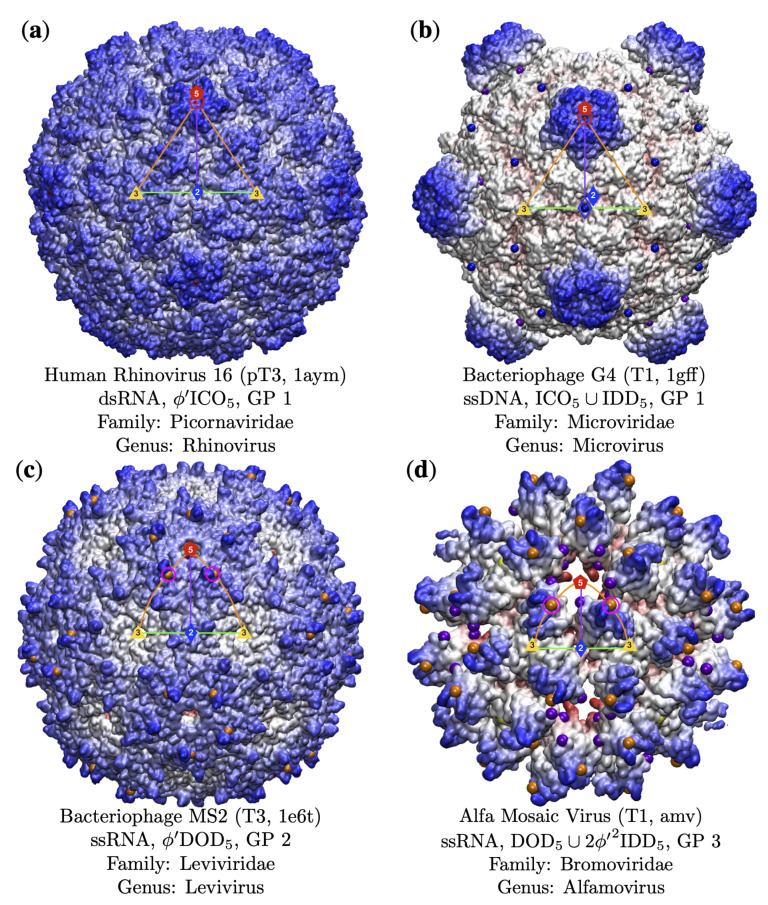
Examples of capsids with gauge points (GP) 1 to 3. The gauge points which correspond to the key structural protrusions are shown in pink circles. The Triangulation number, pdb id, genome composition, point array classification [3]. (**a**) Human Rhinovirus [36] has a structural protrusion on the 5-fold axes (GP 1). There is another protrusion along the 5–3 GC; however, none of the point arrays corresponding to these protrusions fit the virus capsid well [3]. (**b**) Bacteriophage G4 [37] uses GP 1 for its protrusions. GP 21 was also a possible fit; however, none of the point arrays associated with this gauge point were a good fit for the capsid. (**c**) Bacteriophage MS2 [38] has surface loops at GP 2, as shown. Previous experimental work showed that while these loops do not appear structural in nature, changes to the amino acids adjacent to GP 2 was nearly impossible [3,20,21]. As all of the proteins in MS2 are chemically identical, this restriction to modification extends to all protrusions of MS2 pre-assembly. (**d**) Alfa Mosaic Virus [39] uses GP 3 for its protrusions and has remarkable agreement (6 radial levels, [3] with the double point array ${\mathrm{DOD}}_{5}\cup 2\phi^{\prime }{\mathrm{IDD}}_{5}$ and with an RMSD of 1.6 Å.

**Figure 8:**
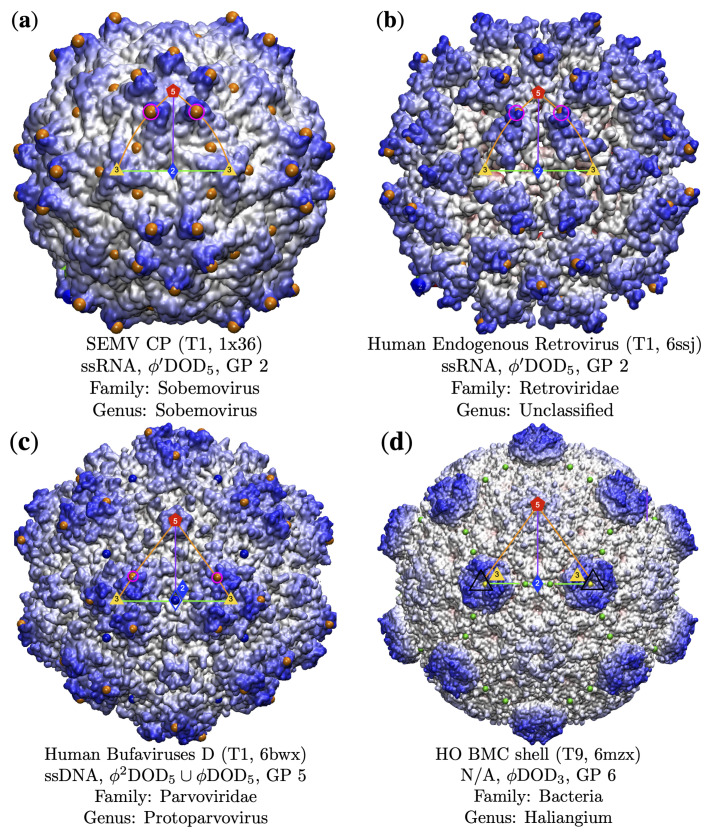
Gauge points along the 5–3 arc (GP 1 to 6). Gauge points which correspond to the key structural protrusions are encircled in pink. Gauge points 2 and 3 are dominated by RNA capsid protrusions (84.6% and 80.0%, respectively). (**a**) Sesbania Mosaic Virus [40] is GP 2 but it also has protrusions near the 3-fold axes; however, these locations are over 5Å below the ones at GP 2, and do not correspond to any point arrays that fit the capsid. We can see that despite using the same gauge point, these two capsids look quite distinct. (**b**) Human Endogenous Retrovirus [41] also uses GP 2, though it looks quite distinct from SEMV, with relatively large openings along the 3–2–3 arc. (**c**) Human Bufaviruses D [42] uses GP 5 for its protrusion. Note the valley between proteins resulting in a lower surface along the 3–2–3 arc. (**d**) An icosahedral bacterial micro-compartment [34] which uses GP 6, only one of two structures in our library to do so. To the eye, this structure is clearly distinct from all others in this paper and we speculate that it is advantageous for viruses to not present as bacterial micro-compartments.

Gauge point 1 is the 5-fold axis and is used by DNA and RNA viruses. Most dsDNA viruses use GP 1 (20 of 42 capsids), accounting for 20 of its 38 dsDNA protrusions located here. We found 13 capsids which were T16 and larger were only composed of dsDNA. There are 10 capsids not shown in Table 3, pT21(2), pT25(2), pT27, T28d, pT31, T43, pT169, T169, which all used gauge point 1 or 21. Moving from the 5-fold axes along the great circle to the 3-fold axes (GP 2 to GP 6), we find that GP 2 is dominated by RNA, 11 of 13 viruses (84.6%). We find that GP 3 is also dominated by RNA, 12 of 16 viruses (75.0%). We find that GP 4 was barely used, of the four viruses that did, two of them also had protrusions elsewhere. Gauge point 5 was dominated by ssDNA, 8 of 11 viruses (72.7%); see Figure 7 and Figure 8. Lastly, gauge point 6 seems to be largely unused by icosahedral capsids. While some adenoviruses are known to have trimeric bundles on the 3-fold axes, we did not have access to these structures arranged with icosahedral symmetry. Curiously, the best-fitting protrusion at the 3-fold axis was a bacterial micro-compartment [34], which does not resemble any other capsids we studied. We postulate that there could be an interaction with 3-fold protrusions and viruses that is being avoided. The locations of the protein protrusions near the 3-fold axes also allow for gauge point 5 in principle; however, none of the capsids which correspond to point were a good fit due to point arrays penetrating protein volumes. This indicates there is something substantially different about this structure and other viral capsids. The only virus we found with a structurally significant 3-fold protrusion was a member of the Reoviridae family [35], which also had a protrusion at GP 3. The best-fitting point array (GP 3) had an RMSD of 2.1 Å, which was 0.5 Å better than the 3-fold array with RMSD of 2.6 Å, and by our prior classification schemes [3] would be considered the best array; however, we have included it here due to it being unusual. The only result that used gauge point 6 was the bacteria compartment 6mzx [34].

#### 3.3.1. T7d Viral Capsids

We initially had difficulty finding the best-fitting point arrays for the T7d Papillomaviridae (5kep [43], 5keq [43], 3iyj [44]) and Polyomaviridae (1sva [45], 6gg0 [46]) capsid families [2], which includes SV40. Each of these capsids are composed entirely of pentamers, resulting in 360 capsid proteins instead of the expected 420 (T7). However, each of these capsids have at least one viral protein found at the 5-fold axes, inside or beneath the pentamers located on 5-fold axes, though this protein is not arranged with global icosahedral symmetry [45,47]. The coordinates for these proteins are not found in their pdb coordinates. However, when we take these into account, each of these 5 capsids are GP 1. It is possible that Bovine Papillomavirus (3iyj [44]) and BK Polyomavirus-Like Particle (6gg0 [46]) could turn out to have structural protrusions at GP 4, as some of the best-fitting point arrays we found were located here; however, none of these point arrays made contact with all the capsid proteins. It is possible that including more coordinate data from the genomes could offer additional point array fits. For the analysis of 6gg0, we fit this capsid neglecting the neutralizing antibody, though we plan to investigate the induced conformation changes in future work.

#### 3.3.2. Parvoviradae Family

**Figure 9:**
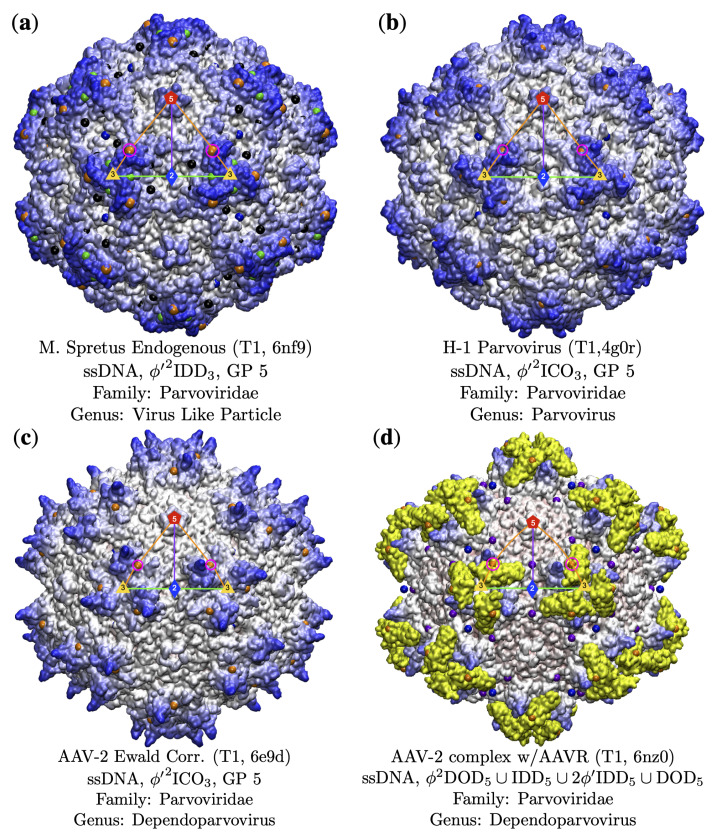
The Parvoviridae Family are non-enveloped T1 capsids with ssDNA that infect a wide range of hosts from vertebrates and invertebrates [48] and all use gauge point 5, making up 8 of the 11 viruses at this location. As you can see in these images, their surfaces vary; however, they all use the exact same gauge point 5 for their structural protrusions. (**a**) M. spretus is a VLP which was discovered as an endogenous viral element. (**b**) The H-1 parvovirus capsid has biomedical applications [50]. (**c**) Adeno-Associated Virus (AAV2) has protrusions that are slightly shifted off GP 5 and was found using cryoEM [51]. No other gauge points and associated point arrays fit this structure. (**d**) Adeno-Associaed Virus in-complex with its cell receptor AAVR, shown in yellow [52]. This combined structure still used GP 5; however, we see that the internal arrangement of constraints has shifted outer point arrays from $\phi^{{}^{\prime }2}{\mathrm{ICO}}_{3}$ to $\phi^{{}^{\prime }2}{\mathrm{DOD}}_{5}$, which is the sister array at GP 5 with one interior radial-level difference [3].

The Parvoviridae Family are non-enveloped T1 capsids with ssDNA that infect a wide range of hosts from vertebrates and invertebrates [48] and all use gauge point 5, making up 8 of the 11 viruses at this location. In Figure 9, you can see that while the surfaces vary, they all use the same locations for their protrusions. One fascinating member of this family is M. spretus, a VLP [49] that was discovered as an endogenous viral elements incorporated in Mus spretus, an Algerian mouse. Its genetic similarity to other parvoviruses indicate that its less than 2 million years old. We note that this reconstituted capsid still uses GP 5, indicated this location is likely of great importance to parvoviruses, perhaps critical to the family overall. The H-1 parvovirus capsid has biomedical applications, and a genome-free VLP has been developed as an antitumor gene delivery vector [50]. Not all members of this family have great point arrays fits, as in the case of Adeno-Associated Virus (AAV2). These protrusions are slightly shifted off GP 5, so that the gauge point rests at the base of them. However, this is still the best-fit array considering all other arrays available. The AAV2 structure was determined by cryoEM using several advancements in techniques, including per-particle CTF refinement and corrections for Ewald sphere curvature, to improve resolution [51]. We intend to study this and related to structures in the future to determine the source of the deviation of the gauge point from the protrusions. We also examined Adeno-Associaed Virus in complex with its cell receptor AAVR [52]; see Figure 9. Here, the complexed structure still used GP 5; however, we found that the internal arrangement of constraints shifted outer point arrays from $\phi^{{}^{\prime }2}{\mathrm{ICO}}_{3}$ to $\phi^{{}^{\prime }2}{\mathrm{DOD}}_{5}$ which is the sister array at GP 5 with one interior radial-level difference [3]. These additional three interior point arrays represent other internal structure changes allowed by this complexed structure, which we therefore expect this complex to be more stable.

### 3.4. Gauge Points 7 to 17: RNA Viruses Only

**Figure 10:**
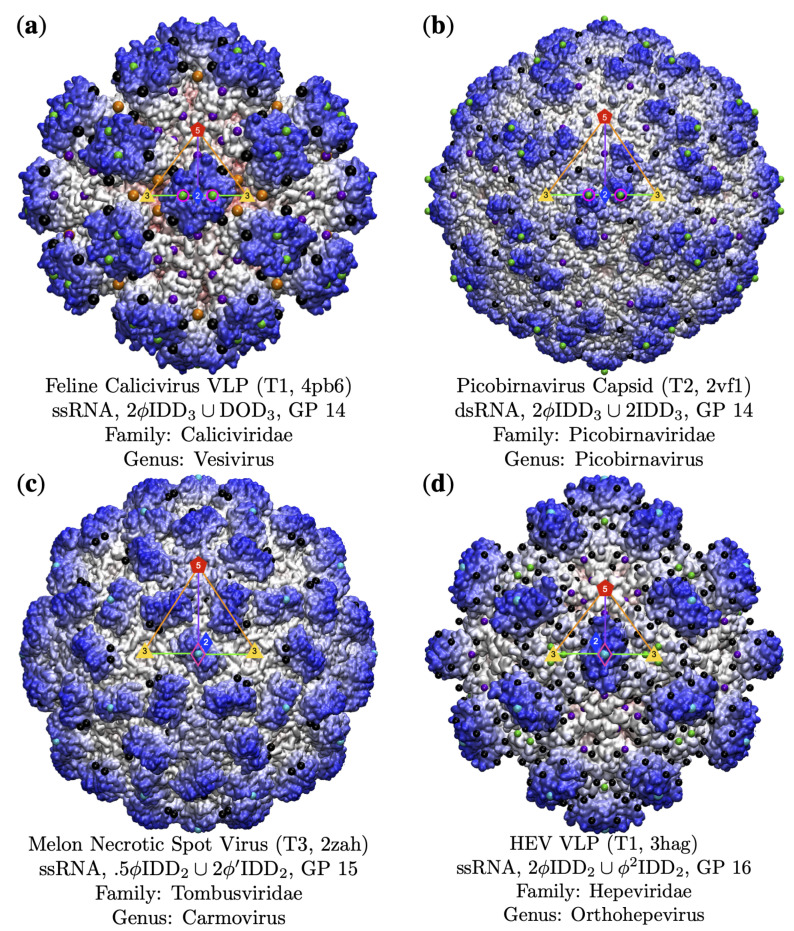
Gauge points 7 to 17 are exclusively RNA viruses. The capsid surfaces are diverse and their protrusions are well located on the gauge points. (**a**) Feline Calicivirus [53] is a VLP with protrusions that flair up near GP 14. (**b**) Picorbirnavirus is a T2 capsid [54] which also uses GP 14, though its surface differs considerably from Feline Calicivirus. (**c**) Melon Necrotic Spot Virus [55] has a structural protrusion at GP 15. There is another protrusion at GP 3; however, none of the associate point arrays fit the capsid well as well. (**d**) Hepatitis E VLP [56] has excellent agreement with its point arrays [3] and uses the 2-fold axes.

**Figure 11:**
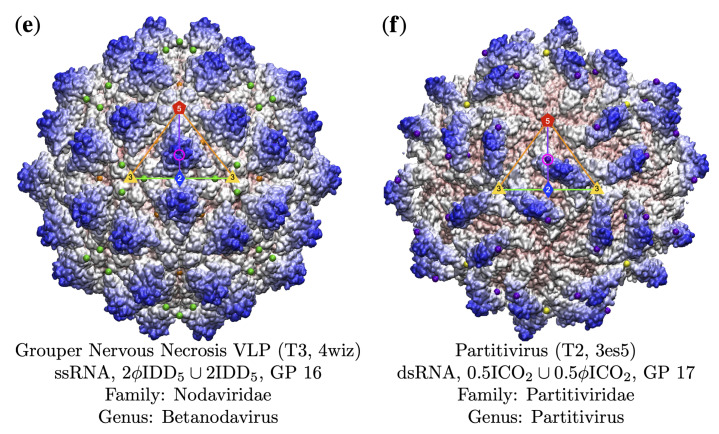
Gauge points 7 to 17 continued. This region is home exclusively to RNA viruses. (**e**) Grouper Nervous Necrosis VLP [57] uses GP 16 that is slightly embedded with the protrusion. Overall, this capsid does not have a great fit with the point arrays, as can be seen with the floating green points. (**f**) Partitivirus [58] has an unusual protrusion that connects to the viral surface in the bulk region; however, the edge of it intersects the 5–2 arc at GP 17.

There are few virus capsids with protrusions along the 3–2–3 arc of the AU, GP 7 to 15; see Figure 5 and Figure 6. Most of the viruses in this region use the 2-fold axes for their protrusions. It is possible that having protrusions in this region lead to smaller capsid volumes, making it more difficulty for DNA packing. Overall, we found that GP 7 to 17 are only RNA viruses (BC IV and BC V), accounting for 28% of all RNA viruses in our library (22 of 29). Gauge point 7 to 14 is sparsely populated, with 7 of 79 RNA viruses. Gauge point 15 to 17 had 15 viruses. The only exception was the T3 HBV capsid, which has a protrusion at the 2-fold axis (GP 15); however, this form of the capsid is unable to fit the DNA genome inside. It is interesting to note that at this stage of assembly, HBV contains pre-genomic RNA [32,33]. See Figure 10 and Figure 11 for examples of viruses along GP 7 to 17. We also note that no viruses were found that use GP 8, 9 or 11.

### 3.5. Gauge Points 18 to 21

**Figure 12:**
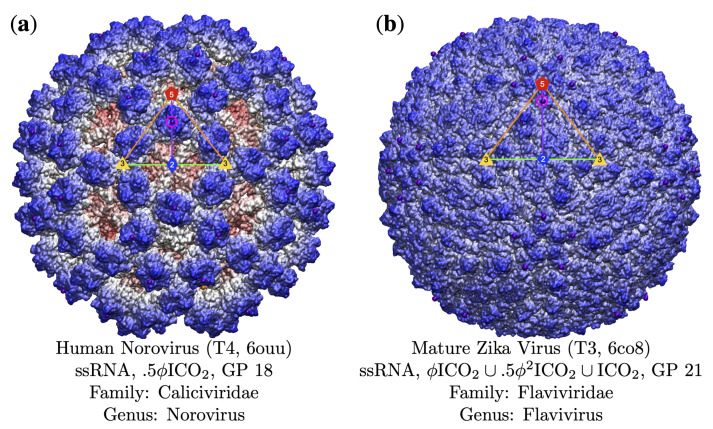
Gauge points 18 to 21. (**a**) Human Norovirus [59] is a T4 capsid with two protruding features; however, only the protrusion at GP 18 has an associated point array that fits the capsid well. (**b**) The Mature Zika Virus [60] has the some of the least differentiated protrusions out of all the viruses in our database. We initially identified protrusions at GP 1, 3, 8, 10, and 21. However, only the point arrays with gauge point 21 shown above had low RMSD scores (1.6Å with GP 21 arrays vs. 3.2Å with GP 8 arrays).

We now turn our attention to the rest of the arc between the 5- and 2-fold axes; see Figure 12. Gauge points 18 and 19 are sparsely populated, with 6 viruses each and a mixture of RNA and DNA composition, see Table 4, Figure 5 and Figure 6. Gauge point 20 is dominated by RNA viruses, 10 of 14 capsids. Finally, GP 21 is equally used by DNA and RNA viruses. This is also the second largest location for dsDNA capsids with 8 of 42 being found here, in-total GP 1 and GP 21, account for 28 of 42 dsDNA capsids. There are also four viruses with T21 or larger architecture that use GP 21; see Table 3.

## 4. Conclusions

Our work shows that Viral Phrenology, the study of spherical capsid protrusions to deduce the genomic composition of a virus, is a useful tool in understanding the structure and assembly of viruses. While the importance of protrusions to infection has been long established, the patterns and rules they obey is just now being revealed. We show that gauge points and their associated point arrays are an important compliment to the traditional Triangulation number and Baltimore Classification of viruses. In total, we provide 21 visual depictions of viral capsids and their associated gauge points, showing the wide range of applicability of these methods. The implications and utility of this work are broad, especially when considering potential modifications of viruses and designing new virus-like particles (VLPs). While the reason for viruses adhering to point arrays are still poorly understood, the rules and patterns which viruses clearly follow are becoming more apparent.

Point arrays continue to be an important tool for understanding viruses, from knowing where it is permissible or not to substitute or modify surface features, to helping explain differences in vibrational properties of chemically identical proteins, to understanding binding affinity on surfaces [3]. Now we see a clear connection to genomic composition as well, which should be considered when modifying viral surfaces and designing new VLPs. The origins of these restrictions is not yet clear, it could very well be evolutionarily fixed, as is possible in the Parvoviradae or Papillomaviridae families.

It is speculated that dsRNA viruses evolved from positive-sense single-strand RNA viruses which then lead to negative-sense single-strand RNA viruses [61]. This could account for each genomic type using all of the gauge points in similar numbers. There is also reason to believe that protrusions along the 2–3–2 section of the AU will lead to smaller capsid volumes than DNA capsids could use, though more study is needed. This smaller volume could explain why the T4 HBV capsid can encapsulate the dsDNA genome but the T3 HBV capsid can not. We also believe that these constraints must be related to the overall stability of the virus capsids as a whole, rather than any localized chemistry differences due to the wide range of unrelated capsids, which all have different surface chemistry. However, even if this adherence turns out to be coincidental to some other energetic effects, point arrays still provide a useful and convenient tool to analyze virus capsids.

It is now clear that point arrays, Baltimore Classification and Triangulation number are all communicating different information about the virus structures. It is our belief that understanding point arrays will provide a connection between Triangulation number and Baltimore Classification, as an understanding of the gauge points has lead to an understanding of genomic composition. It is also clear that some T-numbers have a strong connection to certain gauge points, which then limit the potential point array structures.

## Figures and Tables

**Table 1:** Viral Capsid Data Set—We analyzed 135 distinct icosahedral capsids found in the VIPERdb [1]. Here, we present their genomic composition vs. T-number to illustrate the diversity of capsids and their genomes. We note that nearly all of the ssDNA capsids are T1 (87%), and most of the ssRNA capsids (70%) are T3 or pT3. Most of the T3 capsids are composed only of ssRNA (87%). There are 10 capsids not shown here—pT21(2), pT25(2), pT27, T28d, pT31, T43, pT169, and T169—which were all dsDNA genomes. It is also clear that there is a connection between a larger T-number and dsDNA, as T7, T16 and larger capsids were found to only contain dsDNA. This is likely due to the inherent stiffness of the dsDNA molecule requiring a larger capsid to contain it. In summary, knowing the T-number only provides limited information on the genome, as does only knowing the genome provide little information on the T-number. We did not find any T12 or T19 capsids as might be expect based on [25], though pT27 and T169 were present.

Genome	T1	T2	T3	pT3	T4	T7d	T7l	T9	T13	T16	Total
dsDNA	2	1	2	0	2	5	11	0	2	3	28
ssDNA	13	0	1	0	0	0	0	0	1	0	15
dsRNA	7	4	0	1	0	0	0	0	5	0	17
ssRNA	13	0	27	16	4	0	0	1	0	0	61
None	1	0	1	0	1	0	0	1	0	0	4
Totals	36	5	31	17	7	5	11	2	8	3	125

**Table 2:** Minimum Radial Constraints Implied by Gauge Points. Gauge points imply a fairly restrictive set of radial and angular locations. Gauge points not located on the icosahedral symmetry axes can only be met by two point arrays (*), referred to as sister arrays and are identical at all but one radial level; see Figure 4. This restriction is the minimal set, as point arrays can be combined to form larger arrays, though no points may be removed; see [3].

GP	Loc.	Arrays per GP	Radial Levels
1	ICO	3	5
2–5	3–5	2 *	5
6	DOD	4	7 or 8
7–14	2–3	2 *	7–11
15	IDD	11	9–16
16–21	5–2	2 *	5–7

**Table 3:** Gauge Points of Protruding Features vs. T-number. In total, we found 149 structurally significant protruding features within the AU when analyzing the 135 icosahedral capsids data set found in VIPERdb [1]. We found that most viruses have only one significant structural protrusion (90%) and the rest had two significant structural protrusions (10%); no virus has more than two. In general, knowing the location of a structural protrusion does not strongly indicate the T-number. However, there are some exceptions—GP 2 is mainly used by T1 capsids (69%), GP 5 is mostly used by T1 capsids (73%) and the 2-fold axes (GP 2) is primarily T3 (88%). We also found that few viral protrusions (6.5%) were found in the region from the 3-fold axes (GP 6) to the 2-fold axes (G15), though the 2-fold (GP 15) is well populated. There are 10 viruses with T-numbers larger than T16 not shown here, and they all had a single structurally significant protrusion located at the 5-fold axes (GP 1) or along the 5–2 region at GP 21. Capsids with two structural protrusions ranged from from T1 to T13 and included each of the four genome types. Numbers appearing in bold are the large majority T-number at these gauge points. This data can be visualized in Figure 5.

GP	T1	T2	T3	pT3	T4	T7d	T7l	T9	T13	T16	Totals
**1** **(5-fold)**	3		12	4	2	5	6		3		35 (25.2%)
**2**	**9**		3	1							13 (9.4%)
**3**	3	1	3	3	2				3		15 (10.8%)
**4**	1		1	1			1				4 (2.9%)
**5**	**8**		1	1					1		11 (7.9%)
**6** (**3-fold**)		1						1			2 (1.4%)
**7**	1										1 (0.7%)
**8**											0 (0.0%)
**9**											0 (0.0%)
**10**				1							1 (0.7%)
**11**											0 (0.0%)
**12**	1										1 (0.7%)
**13**		1	1								2 (1.4%)
**14**	1	1									2 (1.4%)
**15** (**2-fold**)	1		**7**								8 (5.8%)
**16**	2		1	1							4 (2.9%)
**17**		1	1	1							3 (2.2%)
**18**	1			1	2		1	1			6 (4.3%)
**19**	2			1	1		1			1	6 (4.3%)
**20**	2	1	2	2	1		2		3	1	14 (10.1%)
**21**	4		2	2			2			1	11 (7.9%)
Total	39	6	34	19	8	5	13	2	10	3	139

**Table 4:** Gauge Points of Protruding Features vs. Genome. The genomic compositions of the 149 structural protrusions of the 135 distinct capsids found in the VIPERdb [1]. We found that only RNA viruses are found to have protrusions from gauge point 7 to 17, which we refer to as the “T” region; see Figure 6. There is one notable exception, the aberrant T3 form of HBV (*), which is too small to actually encapsulate its DNA genome [32,33]. We also see that RNA capsids protrusions dominate GP 2 (85%), GP 3 (80%) and GP 20 (71%). Capsids with ssDNA are also clearly dominant at GP 5 (73%).

GP	dsDNA	ssDNA	dsRNA	ssRNA	None	Totals
**1** (**5-fold**)	20	5	1	13	2	41
**2**	1	1	2	9		13
**3**	2	1	4	8		15
**4**	1		1	2		4
**5**		**8**	1	2		11
**6** (**3-fold**)	1				1	2
**7**			1			1
**8**						0
**9**						0
**10**				1		1
**11**						0
**12**			1			1
**13**			1	1		2
**14**			1	1		2
**15** (**2-fold**)	1 *			**7**		8
**16**				4		4
**17**			1	2		3
**18**	2			4		6
**19**	3		1	2		6
**20**	3	1	4	6		14
**21**	8		1	5	1	15
Totals	42	16	20	67	4	149

## Data Availability

Our pdb library list is available in the Appendix A. Any other data is available upon request.

## Abbreviations

The following abbreviations are used in this manuscript:

AU	Asymmetric Unit
BC	Baltimore Classification
GP	Gauge Point
PA	Point Array
TX	Triangulation number X, e.g., T3 or T7.

## Appendix A. Data Set

From the over 600 viral capsids deposited in VIPERdb [1], we constructed a library of 135 capsids. The complete library is found in Table A1.

**Table A1:** Viral Capsid Library—here are the 135 distinct capsid with unique families, genera, Triangulation numbers and lowest resolutions. When there was a tie for lowest resolution, both structures were used. Resolution ties accounted for 6 additional capsids.

Family	Genus	T#	GP	pdb
Adenoviridae	Mastadenovirus	1	19	4aqq
	Mastadenovirus	pT25	21	6b1t
Bacteria	Haliangium	9	6	6mzx
Birnaviridae	Aquabirnavirus	1	7	3ide
	Avibirnavirus	1	12	2df7
	Avibirnavirus	13	3 or 20	1wce
Bromoviridae	Alfamovirus	1	3	amv
	Bromovirus	1	21	1yc6
	Bromovirus	3	5 or 20	1za7
	Cucumovirus	3	20	1f15
Caliciviridae	Lagovirus	3	15	3j1p
	Norovirus	1	16	6ouc
	Norovirus	3	15	6p4j
	Norovirus	3	1	6p4l
	Norovirus	4	18	6ouu
	Vesivirus	1	14	4pb6
	Vesivirus	3	15	2gh8
Chrysoviridiae	Chrysovirus	1	19 or 21	3j3i
Circoviridae	Circovirus	1	20	5j37
Corticoviridae	Corticovirus	pT21	1	2w0c
Cystoviridae	Cystovirus	2	13	4btg
Dicistroviridae	Aparavirus	pT3	3	5lwg
	Cripavirus	pT3	20	1b35
	Triatovirus	pT3	3	5mqc
Flaviviridae	Flavivirus	3	21	6co8
Hepadnaviridae	Orthohepadnavirus	3	3 or 15	6bvn
	Orthohepadnavirus	4	18	6hu4
Hepeviridae	Hepevirus	1	15	3hag
Herpesviridae	Alphaherpesvirinae	16	19	5zap
	Muromegalovirus	16	21	6nhj
	Rhadinovirus	16	20	6b43
Iflaviridae	Iflavirus	pT3	17	5lsf
Lavidaviridae	Sputnikvirus	pT27	1	3j26
Leviviridae	Allolevivirus	3	3	5vly
	Levivirus	1	16	4zor
	Levivirus	3	2	1e6t
	Levivirus	3	2	2bu1
	Unclassified	3	4 or 13	2vf9
Microviridae	Microvirus	1	1	1gff
	Microvirus	1	1	2bpa
Myoviridae	T4virus	13	1	5vf3
Nodaviridae	Alphanodavirus	3	1	4ftb
	Betanodavirus	1	2	4rft
	Betanodavirus	3	16	4wiz
	Unclassified	1	20 or 2	5yl1
	Unclassified	3	17	4nwv
	Unclassified Nodaviridae	3	1	6jjc
Papillomaviridae	Alphapapillomavirus	1	21	1dzl
	Alphapapillomavirus	7d	1	5kep
	Alphapapillomavirus	7d	1	5keq
	Deltapapillomavirus	7d	1	3iyj
Partitiviridae	Partitivirus	2	17	3es5
Parvoviridae	Ambidensovirus	1	5	4mgu
	Bocaparvovirus	1	5	5us7
	Densovirus	1	5	3p0s
	Dependoparvovirus	1	5	6e9d
	Dependoparvovirus	1	5	6nz0
	Erythroparvovirus	1	2 or 3	6nn3
	Parvovirus	1	5	4g0r
	Penstyldensovirus	1	1	3n7x
	Protoparvovirus	1	5	6bwx
	Virus-Like Particle	1	5	6nf9
Phycodnaviridae	Chlorovirus	169	21	6ncl
	Chlorovirus	pT169	1	1m4x
Picobirnaviridae	Picobirnavirus	2	14	2vf1
Picornaviridae	Apthovirus	pT3	3 or 4	1qqp
	Cardiovirus	pT3	19	5cfc
	Cardiovirus	pT3	2	5cfd
	Enterovirus	pT3	1	4q4w
	Enterovirus A	pT3	21	6smg
	Enterovirus F	pT3	5 or 21	6t40
	Heptaovirus	3	1	4qpi
	Heptaovirus	pT3	10	6jhs
	Kobuvirus	pT3	1	5aoo
	Parechovirus	pT3	16	6gv4
	Rhinovirus	pT3	1	1aym
	Senecavirus	pT3	18	3cji
Podoviridae	Bpp1virus	7l	1	3j4u
	Epsilon15-like	7l	20	3j40
	P22-like	7l	20 or 18	2xyy
	P22virus	7l	1	5l35
	Phi29virus	3	2	1yxn
	T7virus	7l	1	3j7w
	Unassigned Autographivirinae	7l	1	2xd8
Polyomaviridae	Betapolyomavirus	7d	1	6gg0
	Polyomavirus	7d	1	1sva
Protogloboviridae	Alphaprotoglobovirus	43	1	6oj0
Reoviridae	Aquareovirus	1	3	5zvt
	Aquareovirus	2	3 or 6	3iyl
	Cypovirus	1	2	3jay
	Cypovirus	2	20	3izx
	Dinovernavirus	1	2	6djy
	Orbivirus	13	5 or 20	2btv
	Orthoreovirus	13	3	2cse
	Phytoreovirus	13	20	1uf2
	Rotavirus	13	3	3iyu
Retroviridae	Unclassified	1	2	6ssj
	Unclassified	3	21	6ssm
Saccharomycetals	Saccharomyces	9	18	6r24
Sarthroviridae	Macronovirus	1	2	6jja
Satellites	Papanivirus	1	18	5cvz
Secoviridae	Comovirus	pT3	1	5fmo
	Nepovirus	pT3	20	2y26
Siphoviridae	Cyanophage Mic1	13	1	6j3q
	Lambdavirus	7l	1	1ohg
	Oshimavirus	7l	21	6o3h
	P23 Virus	7l	19 or 21	6i9e
	Phietavirus	4	1	6b23
	Phietavirus	7l	4	6b0x
	Tequintavirus	13	1	6omc
	Unclassified Siphovirus	7l	1	6r3a
Sobemovirus	Sobemovirus	1	2	1x36
	Sobemovirus	3	1	1ng0
Sphaerolipoviridae	Alphasphaerolipovirus	28d	21	6qt9
Tectiviridae	Tectivirus	pT25	1	1w8x
	Betatetravirus	4	3	2qqp
	Omegatetravirus	4	19 or 3	3s6p
Thermococcales	Pyrococcus	3	1	2e0z
Togaviridae	Alphavirus	4	20	6imm
Tombusviridae	Aureusvirus	3	15	6mrl
	Carmovirus	3	15	2zah
	Dianthovirus	3	15	6mrm
	Machlomovirus	3	1	3jb8
	Necrovirus	3	1	1c8n
	Panicovirus	3	1	4v99
	Tombusvirus	3	3	4llf
Totiviridae	Totivirus	1	4	1m1c
Turriviridae	Alphaturrivirus	pT31	1	3j31
Tymoviridae	Tymovirus	3	1	1ddl
Unclassified	Unclassified	3	1	6izl
Unclassified	Unclassified	4	1	6tap
Unknown	Unknown	pT21	21	5oac
Virtovirus	Tobacco virtovirus 1	1	2	4oq9
Virus-Like Particle	Virus-Like Particle	1	21	6i9g
Virus-Like Particle	Virus-Like Particle	3	1	4pt2
